# Distinct gene signatures of monocytes and B cells in patients with giant cell arteritis: a longitudinal transcriptome analysis

**DOI:** 10.1186/s13075-022-02982-9

**Published:** 2023-01-03

**Authors:** Kotaro Matsumoto, Katsuya Suzuki, Hiroto Yoshida, Mayu Magi, Yoshihiro Matsumoto, Mariko Noguchi-Sasaki, Keiko Yoshimoto, Tsutomu Takeuchi, Yuko Kaneko

**Affiliations:** 1 Division of Rheumatology, Department of Internal Medicine, Keio University School of Medicine, 35 Shinanomachi, Tokyo, Shinjuku-ku Japan; 2 Chugai Pharmaceutical Co. Ltd., 200 Kajiwara, Kamakura, Kanagawa Japan

**Keywords:** Large-vessel vasculitis, Giant cell arteritis, Gene expression

## Abstract

**Background:**

Giant cell arteritis (GCA) is a primary large-vessel vasculitis (LVV) of unknown origin. Its management is a challenge due to the late onset of disease symptoms and frequent relapse; therefore, clarifying the pathophysiology of GCA is essential to improving treatment. This study aimed to identify the transition of molecular signatures in immune cells relevant to GCA pathogenesis by analyzing longitudinal transcriptome data in patients.

**Methods:**

We analyzed the whole blood transcriptome of treatment-naive patients with GCA, patients with Takayasu arteritis (TAK), age-matched, old healthy controls (HCs), and young HCs. Characteristic genes for GCA were identified, and the longitudinal transition of those genes was analyzed using cell-type identification by estimating relative subsets of RNA transcripts (CIBERSORT).

**Results:**

Repeated measures analysis of variance revealed 739 differentially expressed genes among all patients and HCs. Of the 739 genes, 15 were characteristically upregulated and 36 were downregulated in patients with GCA compared to those with TAK and HCs. Pathway enrichment analysis showed that downregulated genes in GCA were associated with B cell activation. CIBERSORT analysis revealed that upregulation of “M0-macrophages” and downregulation of B cells were characteristic of GCA. Upregulation of “M0-macrophages” reflects the activation of monocytes in GCA toward M0-like phenotypes, which persisted under 6 weeks of treatment. Combined treatment with prednisolone and an interleukin-6 receptor antagonist normalized molecular profiles more efficiently than prednisolone monotherapy.

**Conclusions:**

Gene signatures of monocyte activation and B cell inactivation were characteristic of GCA and associated with treatment response.

**Supplementary Information:**

The online version contains supplementary material available at 10.1186/s13075-022-02982-9.

## Background

Giant cell arteritis (GCA) is a systemic inflammatory vasculitis of unknown etiology that occurs in the elderly and causes a wide variety of systemic, neurologic, and ophthalmologic symptoms. The Chapel Hill Consensus Conference 2012 definition [1] classifies GCA as a primary large-vessel vasculitis (LVV), along with Takayasu arteritis (TAK). The clinical characteristics of GCA and TAK are distinct; GCA develops in people over the age of 50 years and is common in Western countries, whereas TAK usually develops in those below 40 years and is common in Japan, China, India, Turkey, Mexico, and South America. Moreover, GCA is related to human leukocyte antigen (HLA)-class I and II alleles, while TAK relates to HLA-class I alleles [2–6].

GCA involves the development of vasculitides in the aorta and its main branches, with predominantly granulomatous infiltrates of T lymphocytes, macrophages, and multinucleated giant cells [7–11]. The pathophysiology of GCA is not yet fully understood; however, the involvement of Th1 and Th17 immune-mediated response and an imbalance of Th17/regulatory T (Treg) have been demonstrated in GCA [9, 12–16]. Besides CD4+ T cells, recent studies identified the molecular profile of circulating CD8+ T cells, monocytes, and neutrophils [17–19].

We have previously reported innate and adaptive immune profiles in vasculitis by broad immunophenotyping and whole blood RNA sequencing [20–25]. A comparison of patients with LVV with healthy controls (HCs) revealed characteristic upregulation of the IL-1 signaling pathway in those with LVV, associated with the extent of disease and poor prognosis, suggesting an association between the pathogenesis of LVV and innate inflammation [20]. In these analyses, however, GCA and TAK were examined together; thus, the individual characteristics of their respective molecular signatures are still unclear. In this study, we performed whole blood RNA sequencing in detail, focusing on patients with GCA.

## Patients and methods

### Study population

To explore the key molecules characteristic of patients with GCA, we used whole blood RNA sequencing data from treatment-naive patients with GCA and TAK and age-matched HCs, described elsewhere [20]. Whole blood samples were collected from patients with GCA and TAK at Keio University Hospital between August 2013 and May 2019 who met the respective international classification criteria [26, 27]. Samples were obtained from patients during week 0 (at the timing of diagnosis prior to initiating induction therapy), after 6 weeks of treatment at remission following treatment with prednisolone and tocilizumab, and at the time of disease relapse. HCs were confirmed to have no history of autoimmune disease, severe allergic disorders, malignancy, or infection.

This study was approved by the Institutional Review Board of Keio University School of Medicine (approval number #20140479) and conducted in compliance with the Declaration of Helsinki. Written informed consent was obtained from all participants.

### Clinical assessment

Clinical information was obtained from the clinical charts and annual medical check-up reports of the patients. We collected information on age, sex, race, body mass index [28], smoking habits, comorbidities (including hypertension, diabetes mellitus, dyslipidemia, fatty liver disease, chronic kidney disease, polymyalgia rheumatica, inflammatory bowel disease, and aortic regurgitation), duration from onset of symptom to diagnosis, arterial involvement (LV and cranial), disease activity (remission and relapse), and laboratory data (including complete blood count, antinuclear antibody (ANA) positivity (≥ 40 titer), rheumatoid factor (RF) positivity (≥ 15 IU/ml), immunoglobulin G level, erythrocyte sedimentation rate, and C-reactive protein (CRP) level). LV involvement was defined from any of the following radiological examinations: ultrasonography, CT, ^18^F-fluorodeoxyglucose PET-CT, and magnetic resonance imaging. Cranial involvement was defined as abnormal findings of histological and/or radiological examination of the temporal artery. Remission was defined as the absence of clinical symptoms and normal CRP levels maintained for at least 12 weeks [29]. Relapse was defined as the reappearance of vasculitis-related manifestations or exacerbation of imaging findings, requiring an increase in glucocorticoid dose or additional immunosuppressive agents [29].

### Analysis of whole blood RNA-sequencing data

Whole blood RNA-sequencing data [20] were used in this study. Total RNA with an integrity value of > 7 was sequenced using NextSeq 500 (Illumina, Inc., San Diego, CA, USA). In comparing the expression between patients with GCA, TAK, and HCs, transcripts below *P* < 0.05 with a |fold-change| > 1.5, using repeated measures analysis of variance, were considered significantly and differentially expressed. The extracted genes were then analyzed using pathway enrichment and cell-type identification by estimating relative subsets of RNA transcripts (CIBERSORT). Enrichment analysis for gene sets and pathways was performed with Enrichr v3.1 using an R interface to query the biological process 2018 database for enrichments with an adjusted *P* < 0.05 [30, 31]. Data from CIBERSORT were uploaded to the web portal (https://cibersort.stanford.edu/) and the LM22 gene signature, allowing the use of sensitive and specific discrimination of 22 human hematopoietic cell phenotypes [32].

### Statistics

All analyses were conducted using the R statistics package version 3.6.1 (The R Foundation for Statistical Computing, Vienna, Austria) or Prism software version 8.0 (GraphPad, San Diego, CA, USA). Continuous data are expressed as a median (interquartile range), and categorical data as numbers and/or percentages. Descriptive statistics were used to summarize the data. Continuous variables were compared using the Mann–Whitney *U* test. Categorical variables were compared using the chi-squared test. The threshold for statistical significance was set at *P* < 0.05.

## Results

### Baseline characteristics of GCA, TAK, and old and young HCs

Patients with treatment-naive GCA (*n* = 17) and treatment-naive TAK (*n* = 6) and old (age-matched to GCA, *n* = 6) and young HCs (age-matched to TAK, *n* = 6) were enrolled. The baseline characteristics of the patients are summarized in Table 1. All patients with GCA had complicated cranial and/or LV involvement. Comparison between patients with GCA and old HCs and patients with TAK and young HCs showed no significant differences in age, sex, race, smoking habits, body mass index, and comorbidities. The positivity of autoantibodies, including ANA (18% vs. 33%), RF (0% vs. 17%), and serum immunoglobulin G levels (1524 vs. 1844 mg/dl) tended to be lower in patients with GCA than those in patients with TAK, although these differences were not significant.

**Table 1:** Baseline clinical characteristics of GCA, TAK, and old and young HCs

Variable	GCA	TAK	GCA vs TAK	Old HC	Young HC	Old vs Young
	*n* = 17	*n* = 6	*P* value	*n* = 6	*n* = 6	*P* value
Age, years	71 (70–78)	47 (41–57)	**< 0.010**	73 (69–75)	58 (31–59)	**< 0.010**
Male, *n* (%)	8 (47)	3 (50)	0.90	2 (33)	3 (50)	0.56
Race, Japanese, *n* (%)	17 (100)	5 (83)	0.093	6 (100)	6 (100)	-
Body mass index, kg/m^2^	20 (17–23)	23 (18–24)	0.42	23 (21–26)	21 (20–22)	0.17
Smoking history, *n* (%)	7 (41)	3 (50)	0.71	1 (17)	1 (17)	1.0
Comorbidities						
Hypertension, *n* (%)	4 (24)	3 (50)	0.64	2 (33)	0 (0)	0.075
Diabetes mellitus, *n* (%)	3 (18)	0 (0)	0.16	0 (0)	0 (0)	-
Dyslipidemia, *n* (%)	6 (35)	1 (17)	0.38	2 (33)	0 (0)	0.075
Fatty liver disease, *n* (%)	1 (6)	0 (0)	0.43	1 (17)	1 (17)	1.0
Chronic kidney disease, *n* (%)	2 (17)	2 (33)	0.25	1 (17)	0 (0)	0.22
Polymyalgia rheumatica, *n* (%)	7 (41)	0 (0)	**0.022**	0 (0)	0 (0)	-
Inflammatory bowel disease, *n* (%)	0 (0)	2 (33)	**0.015**	0 (0)	0 (0)	-
Aortic regurgitation, *n* (%)	3 (11)	2 (11)	**0.015**	1 (11)	0 (0)	0.22
GCA with positive TAB finding, *n* (%)	10 (59)	-	-	-	-	-
LV involvement, *n* (%)	12 (71)	6 (100)	0.062	-	-	-
Time from symptom onset to diagnosis, weeks	11 (5.1–21)	23 (9.6–47)	0.11	-	-	-
Laboratory tests at diagnosis						
White blood cells, 10^3^ cells/μL	7.0 (6.2−7.9)	8.9 (6.4–9.9)	0.13	-	-	-
Neutrophils, 10^3^ cells/μL	5.2 (4.0−5.9)	6.4 (4.6–7.4)	0.17	-	-	-
Lymphocytes, 10^3^ cells/μL	1.2 (1.1−1.5)	1.9 (1.0–2.1)	0.20	-	-	-
Monocytes, cells/μL	432 (336−520)	496 (349–577)	0.55	-	-	-
Eosinophils, cells/μL	94 (73−159)	115 (58–263)	0.70	-	-	-
Hemoglobin, g/dL	11 (9.4−12)	11 (10–12)	0.80	-	-	-
Platelets, 10^4^ /μL	36 (27−40)	38 (30–55)	0.53	-	-	-
ANA positivity, *n* (%)	3 (18)	2 (33)	0.44	-	-	-
RF positivity, *n* (%)	0 (0)	1 (17)	0.093	-	-	-
IgG, 10^3^ mg/dL	1.5 (1.3−1.6)	1.7 (1.5−2.5)	0.11	-	-	-
ESR, mm/h	116 (78−131)	87 (63–116)	0.14	-	-	-
CRP, mg/dL	4.5 (2.1−7.6)	3.9 (1.6–9.4)	0.60	-	-	-

Continuous data were expressed as a median (interquartile range) and categorical data as numbers (percentages). Continuous variables were compared using the Mann–Whitney *U* test and categorical variables using the chi-squared test. Values in bold are statistically significant (*P* < 0.05). *GCA* giant cell arteritis, *TAK* Takayasu arteritis, *HC* healthy control, *TAB* temporal artery biopsy, *LV* large vessel, *ANA* antinuclear antibody, *RF* rheumatoid factor, *ESR* erythrocyte sedimentation rate, *CRP* C-reactive protein

### Identification of characteristic molecular profiles for GCA, TAK, and old and young HCs

**Fig. 1:**
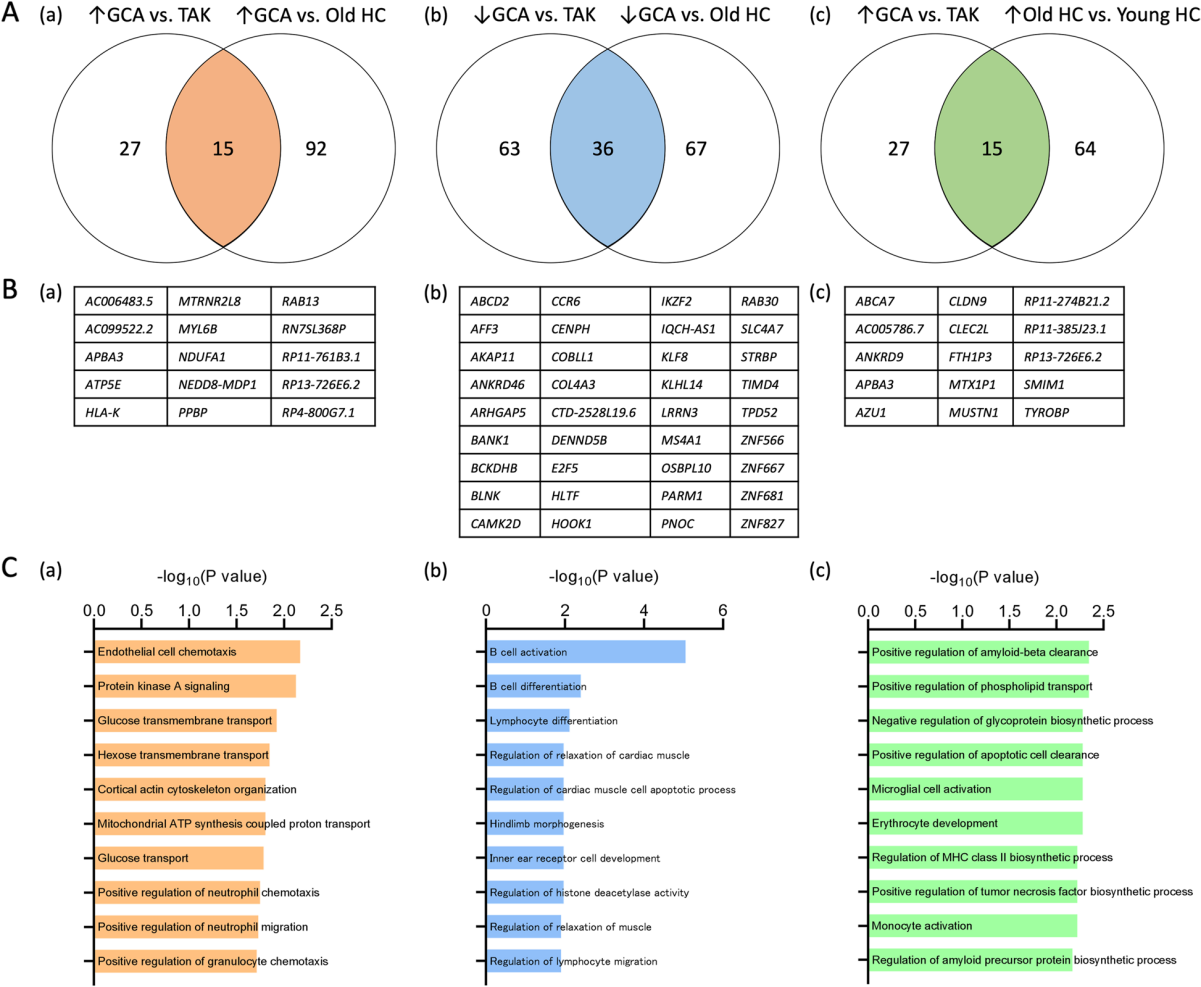
Differentially expressed genes in GCA, TAK, and old and young HCs. A Numbers and B lists of (a) upregulated and (b) downregulated genes in GCA compared with that in TAK and old HCs and (c) upregulated genes in older subjects (GCA vs. TAK and old vs. young HCs). C Top 10 (a) upregulated and (b) downregulated pathways in GCA compared with that in TAK and old HCs and upregulated pathways in older subjects (GCA vs. TAK and old vs. young HCs). Orange, blue, and green: *P* < 0.05. GCA, giant cell arteritis; TAK, Takayasu arteritis; HC, healthy control

A comparison of gene expression data among all patients and HCs revealed 739 differentially expressed genes among GCA, TAK, old HCs, and young HCs (*P* < 0.05, |fold-change| > 1.5). We then compared the expressed genes between GCA and TAK and GCA and old HCs and identified 15 upregulated (Fig. 1A-a, B-a) and 36 downregulated genes (Fig. 1A-b, B-b) characteristic of GCA. Pathway enrichment analysis showed that pathways associated with endothelial cell chemotaxis, protein kinase A signaling, and glucose transmembrane transport were overrepresented among the 15 upregulated genes (Fig. 1C-a), and pathways associated with B cell activation were included among the 36 downregulated genes (Fig. 1C-b). Next, we compared gene expression between GCA and TAK and old and young HCs and identified 15 genes characteristic of aging (Fig. 1A-c, B-c). Pathways associated with positive regulation of amyloid-beta clearance and phospholipid transport were overrepresented among the 15 upregulated genes (Fig. 1C-c) but did not overlap with the identified GCA-characteristic genes. Among patients with GCA, we further compared gene expression between those with and without PMR and those with (LV-GCA) and without LV involvement (cranial-GCA), although there were no specific findings (not shown).

### Assessment of GCA, TAK, and old and young HCs using CIBERSORT analysis

**Fig. 2:**
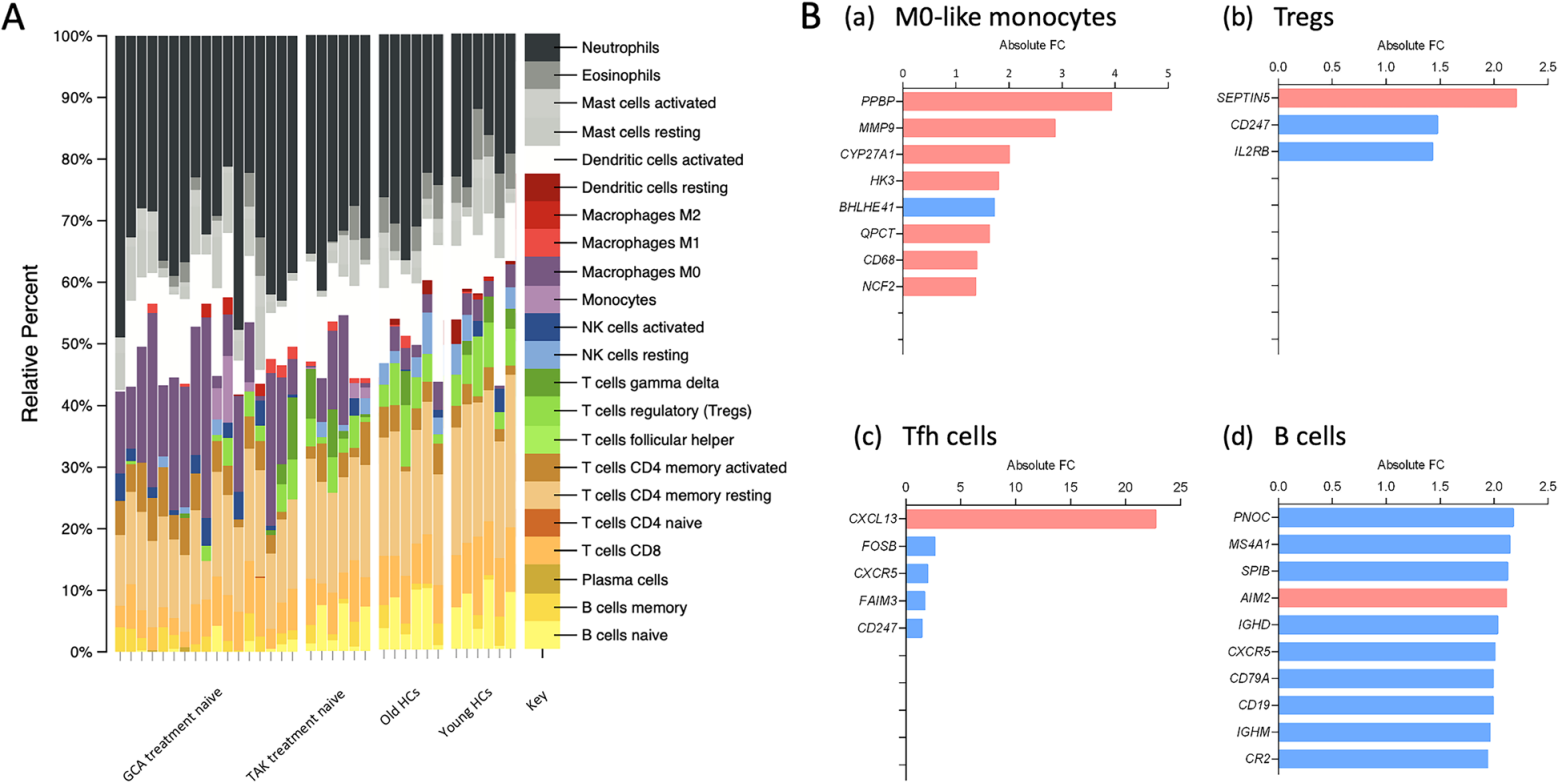
Relative immune cell subsets using CIBERSORT analysis in LVV and HCs. Results of the CIBERSORT analysis in LVV and HCs. **A** Patients with treatment-naive GCA, those with treatment-naive TAK, and old and young HCs. **B** The top 10 dysregulated genes in GCA defining M0-like monocytes, Tregs, Tfh cells, and B cells. Red, upregulated; blue, downregulated genes. LVV, large-vessel vasculitis; GCA, giant cell arteritis; TAK, Takayasu arteritis; HC, healthy control; CIBERSORT, cell-type identification by estimating relative subsets of RNA transcripts; NK, natural killer; FC, fold-change

We performed CIBERSORT to identify the relative immune cell subsets in treatment-naive patients with GCA (*n* = 17), treatment-naive patients with TAK (*n* = 6), old HCs (*n* = 6), and young HCs (*n* = 6). The gene expression related to “M0 macrophages” (M0-like monocytes) was upregulated and that related to Tregs, follicular helper T (Tfh) cells, and B cells was downregulated in treatment-naive patients with GCA compared to that in treatment-naive patients with TAK or HCs (Fig. 2A). We compared patients with GCA and age-matched HCs to focus on genes defining M0-like monocytes, Tregs, Tfh cells, and B cells in the CIBERSORT. Absolute FCs are calculated with an adjusted *P* < 0.1. For each subset, the top 10 dysregulated genes are shown (Fig. 2B). Regarding genes defining M0-like monocytes, gene expression of *PPBP*, *MMP9*, *CYP27A1*, *HK3*, *QPCT*, *CD68*, and *NCF2* were upregulated, while that of *BHLHE41* was decreased in patients with GCA compared to HCs (Fig. 2B-a).

### Longitudinal transcriptome profiles for GCA using CIBERSORT analysis

**Fig. 3:**
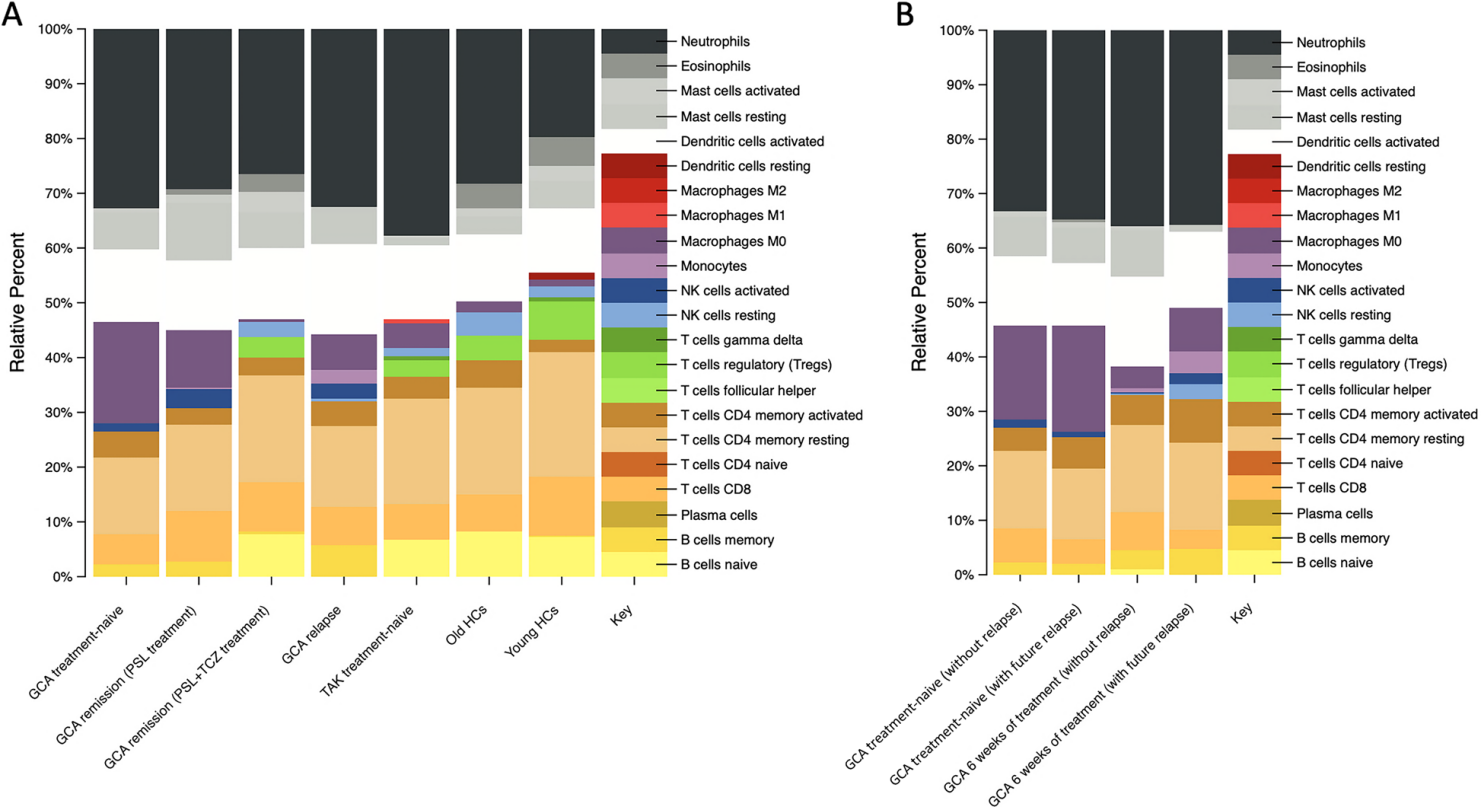
Longitudinal analysis for the proportion of estimated subsets in LVV. The average proportion of estimated subsets was calculated using CIBERSORT analysis. **A** Patients with treatment-naive GCA, remission with prednisolone monotherapy, remission and relapse with prednisolone and tocilizumab treatment, treatment-naive patients with TAK, and HCs. **B** Treatment-naive patients with GCA after 6 weeks of treatment without relapse and with future relapse. LVV, large-vessel vasculitis; GCA, giant cell arteritis; TAK, Takayasu arteritis; HC, healthy control; PSL, prednisolone; TCZ, tocilizumab; CIBERSORT, cell-type identification by estimating relative subsets of RNA transcripts

We also compared the proportion of estimated immune cell subsets per group (Supplementary Table 1): treatment-naive (*n* = 17), remission under prednisolone treatment (*n* = 8), remission under prednisolone and tocilizumab treatment (*n* = 3), and relapse after remission during an observational period of 60 weeks (*n* = 5). The mean indices of the estimated subsets are shown in Fig. 3A. The dysregulated gene signatures before treatment returned to a state similar to that of HCs after attaining remission with treatment and reappeared at relapse. Combined treatment with prednisolone and tocilizumab normalized the levels of dysregulated genes associated with M0-like monocytes, Tregs, Tfh cells, B cells, and imbalance of activated/resting NK cells in GCA more efficiently than prednisolone monotherapy (Fig. 3A). Later, we compared samples from treatment-naive patients with GCA who did not relapse (*n* = 12) with those who relapsed later (*n* = 5) and samples from patients 6 weeks after treatment initiation who did not relapse (*n* = 8) with those who relapsed later (*n* = 5) and found that initial gene signatures were associated with treatment response. Although gene signatures associated with M0-like monocytes were high before treatment irrespective of future relapse, they continued to remain high 6 weeks after treatment in patients with future relapse compared to the apparent decrease 6 weeks after treatment in patients without relapse (Fig. 3B).

## Discussion

Elucidating the differences in pathophysiology between GCA and TAK remains a challenge, despite their distinct differences in clinical features. In this study, we performed a transcriptome analysis and identified the molecular biological features of GCA compared with TAK and age-matched HCs. Longitudinal assessment of transcriptome data identified persistent myeloid dominance in GCA, consistent with a previous article by Sleen et al. using a longitudinal leukocyte immunophenotyping [33]. Relative immune cell subsets obtained from the CIBERSORT in this study revealed that the persistent myeloid dominance was due to persistent monocyte activation. We also identified the activated monocytes in GCA changed toward M0-like phenotypes at the transcriptome level. Further longitudinal assessment revealed that dysregulated gene expression returned to a healthy state under combination treatment with prednisolone and tocilizumab.

Monocyte/macrophage critically contributes to the initiation of vascular inflammation and multinucleated giant cell formation in patients with GCA and TAK [7, 8]. Although macrophage/multinucleated giant cell invasion is a common characteristic of GCA and TAK, our study showed a larger number of circulating M0-like monocytes. M0-like monocytes in GCA were represented by upregulation of *MMP9* (matrix metalloproteinase 9), which was consistent with a previous article [34]. Numbers and phenotypes of macrophages altered during aging. A larger number of circulating M0-like monocytes may suggest the involvement of inflammaging in GCA [35–37]. Furthermore, the gene signature associated with M0-like monocytes remained high in patients with future relapses in the early phase of treatment and even in remission. Considering that the IL-1 signaling pathway was upregulated in patients with future relapse compared to those without [20], chronic monocyte/macrophage activation may induce low-grade inflammation via cytokine production and subsequently contribute to future relapse. Suppression of residual inflammation may preclude disease progression in patients with GCA.

Contrary to increased M0-like monocytes, gene expression associated with Tregs, Tfh cells, and B cells was decreased in GCA. This is consistent with the previous flow cytometry analyses showing low numbers of Tregs and B cells in GCA [21, 33]. Regarding B cells, the circulating number of naive B, memory B, CXCR3+ switched memory B, and CXCR5+ switched memory B cells was lower in GCA than those in HCs [21, 38]. Considering that the decreased level of B cells returned to normal after treatment in the CIBERSORT analysis, investigation of whole blood transcriptome can provide the level of B cells associated with disease activity. In our study, gene expression of *CCR6* was downregulated in GCA. Since *CCR6* widely expresses on memory T and B cells [32, 39, 40], downregulation of *CCR6* possibly reflects homing and its organization. The ratio of activated/resting NK cells was also decreased in treatment-naive patients with GCA, which returned to normal after treatment. However the phenotype of NK cells in GCA is understudied [41], an imbalanced activated/resting NK cells may contribute to the pathogenesis of GCA.

Recent clinical trials of tocilizumab have revealed that therapies targeting IL-6 can effectively reduce GCA relapse and decrease prednisolone dosage [29]. Tocilizumab demonstrates a clinically meaningful benefit with a specific effect of IL-6 inhibition on antibody-producing B cells [42]. Recent studies have also revealed that tocilizumab affects the proportion and function of Tregs, which may explain its efficacy [13–16]. Tocilizumab also affects the number of Th1, Th17, and Tfh cells, suggesting that suppression of these cells may be another therapeutic effect of tocilizumab [21]. Our study demonstrated that tocilizumab in combination with prednisolone dramatically normalized the levels of dysregulated genes associated with M0-like monocytes, Tregs, Tfh cells, B cells, and imbalanced activated/resting NK cells in GCA, the effect of which was greater than that of prednisolone monotherapy. The results were consistent with a study in patients with rheumatoid arthritis, showing that tocilizumab treatment normalized the molecular signature at whole blood transcriptome levels to a greater extent than treatment with methotrexate or a tumor necrosis factor inhibitor [43]. Tocilizumab may induce deeper molecular remission in various inflammatory connective tissue diseases via IL-6 inhibition.

Non-immune-mediated gene ontology terms associated with protein kinase A signaling, glucose and hexose transmembrane transports, cortical actin cytoskeleton organization, and mitochondrial ATP synthesis-coupled protein and glucose transports were all upregulated, while regulation associated with relaxation of cardiac muscle and cardiac muscle cell apoptotic process, hindlimb morphogenesis, inner ear receptor cell development, histone deacetylase activity, and muscle relaxation was downregulated in GCA. These may reflect the abnormalities of cellular metabolisms and cell development in GCA [14, 44, 45], although further investigations are needed.

Our study had several limitations. First, the result was based on a small sample. Second, different treatment regimens were not standardized, and this could affect cell composition. Third, analyses were biased because patients with TAK were relatively old in our cohort. Fourth, the results of the CIBERSORT analysis were different from the percentage of circulating immune cell subsets because the treatment was designed to assess the leukocyte deconvolution from bulk tumors. For these reasons, additional evidence is required to strengthen our findings to make prediction possible for disease relapse in GCA. Allowing for these limitations, the results from whole blood transcriptome analysis have provided valuable insights into the pathophysiology of GCA, particularly regarding molecular signature levels of B cells and monocytes, and illustrated the effect of tocilizumab in normalizing residual molecular signatures. Further studies are needed to confirm our results and clarify the pathophysiology of GCA.

## Supplementary Information

**Additional file 1: Supplementary Table 1.** Treatment regimens at sample acquisition in GCA.

## Data Availability

Data are available on reasonable request. Transcriptome data are available on request to Dr. KM: aa615119@keio.jp.

## Abbreviations

LVV: Large-vessel vasculitis
GCA: Giant cell arteritis
TAK: Takayasu arteritis
HC: Healthy control
CIBERSORT: Cell-type identification by estimating relative subsets of RNA transcripts
HLA: Human leukocyte antigen
IFN-γ: Interferon-gamma
IL: Interleukin
ANA: Antinuclear antibody
RF: Rheumatoid factor
CRP: C-reactive protein
NK: Natural killer
Treg: Regulatory T
Tfh: Follicular helper T
