# Supplementary material for: Distinct gene signatures of monocytes and B cells in patients with giant cell arteritis: a longitudinal transcriptome analysis
Source: Arthritis Res Ther. 2023 Jan 3;25:1. doi: 10.1186/s13075-022-02982-9 (PMC9809009; doi:10.1186/s13075-022-02982-9)
Supplement: Supplementary file 1 — Additional file 1: Supplementary Table 1. Treatment regimens at sample acquisition in GCA. [file 13075_2022_2982_MOESM1_ESM.docx]

Supplementary Table 1. Treatment regimens at sample acquisition in GCA

| Variable | 0 wk | 6 wk | Remission | Remission | Relapse |
| --- | --- | --- | --- | --- | --- |
|  |  |  | (PSL) | (PSL+TCZ) |  |
|  | n = 17 | n = 13 | n = 8 | n = 3 | n = 5 |
| PSL |  |  |  |  |  |
| Duration after initiation, wk | - | 5.9 (4.2−6.4) | 52 (51−58) | 21 (17−23) | 17 (14−42) |
| Dosage, mg | - | 30 (23−40) | 5.0 (4.3−6.8) | 9.0 (8.0−12) | 10 (5.0−16) |
| TCZ |  |  |  |  |  |
| Duration after initiation, wk | - | 3.1 (n = 1) | - | 13 (8.0−16) | 2.0 (n = 1) |
| Routes of administration | - | DIV (n = 1) | - | SC | SC |
| Dosage, mg | - | 8 mg/kg/4 wk (n = 1) | - | 162 mg/2 wk (n = 2) | 162 mg/2 wk (n = 1) |
|  |  |  |  | 162 mg/wk (n = 1) |  |

Treatment regimens of PSL and TCZ, including dosages, routes of administration, and duration. Wk, week; PSL, prednisolone; TCZ, tocilizumab, DIV, intravenous drip; SC, subcutaneous
